# A Question of Graft

**Published:** 1912-06

**Authors:** 


### A Question of Graft

THE editor has recently been asked to denounce a movement, noted as a matter of news in a recent issue, and regarded at the time as a commendable arrangement for the care of unfortunate patients. Without entering into details, we may say that the claim is made of the regular, old-fashioned exploitation of public philanthropy, for private ends. While we are willing to hit from the shoulder, in a question of right and wrong, we do not believe in stirring up quarrels and, in the present instance, the remoteness of the institution attacked and other circumstances, prevent a personal investigation. We are perfectly willing to give space to reasonably brief and temperate discussions of any ethical problem arising in our territory or applying to it.

Would it not be better to deal with this question, which is continually arising and continually fomenting professional discord, on broad lines of scientific prophylaxis? Is not the profession well enough organized at present, to look after its own interests and, incidentally, after the interests of well disposed members who, under older methods, were really innocent tools of designing schemers and who are still, to some degree, the victims of a bad and obsolete system of patronage? Certainly no one would claim that private individuals, privately selected, should benefit by public taxation, nor that charity publicly asked for the discharge of a public duty to the sick and poor, should redound to the profit of a favored few.

Private feuds in the profession, washing of dirty linen in the presence of representatives of the popular press, individual attacks on individuals or selected institutions, do untold harm to the profession. Let us act harmoniously and with a unanimous and sincere determination to formulate proper rules and to apply them impartially, with due regard to ability, experience and demonstration of fitness and devotion, but with fairness to all.

